# Interoception and pain: body–mind integration, rupture, and repair

**DOI:** 10.1097/j.pain.0000000000003515

**Published:** 2025-02-06

**Authors:** Sarah N. Garfinkel, Christopher Eccleston

**Affiliations:** a Institute of Cognitive Neuroscience, University College London, London, United Kingdom; b Centre for Pain Research, University of Bath, Bath, United Kingdom; c Department of Experimental-Clinical and Health Psychology, Ghent University, Ghent, Belgium; d Department of Psychology, The University of Helsinki, Helsinki, Finland

## 1. Introduction

Interoception—how the internal state of the body is represented physiologically and psychologically—offers a new frontier for understanding body–mind integration in the context of pain. Interoceptive science has grown in the last 10 years: from the earlier study of peripheral signalling to new methodologies to quantify bodily representation at distinct hierarchical levels of processing. The time is right for a thorough consideration of body–brain integration in pain that promises to enable novel insights and opportunities for clinical intervention. Interoception can be delineated across multiple levels of pain processing, from the afferent signalling of the body to the motivational attribution of sensation to illness or action.

Each interoceptive dimension has implications for the processing of pain, across conscious and unconscious levels. Merging interoceptive and pain science offers unique opportunity for mechanistic discovery, in particular for the onset and maintenance of high impact chronic pain.^15^ First, we review the field of interoception developed outside of pain, with attention to its recent history and its methods. Second, we review how these methods have been applied in pain and draw them into a platform of shared methods and insights, which can guide a programme of study. For example, we will draw on the few extant studies on interoceptive precision, the neural processing of interoceptive signals, and the nature of afferent signals on pain processing as they may relate to body–mind integration, rupture, and repair. Third, we propose a model of interoception and pain with a focus on different levels of analysis from integration of afferent input driving homeostasis, through to the cognitive architecture of attention to interruption and the function of belief structures around symptom perception.

## 2. Interoception for action

While exteroceptive senses such as vision help us navigate the external world, interoception is an internal sense, relating to the process by which the nervous system senses, interprets, and integrates signals originating from within the body, providing a moment-by-moment mapping of the body's internal landscape across conscious and unconscious levels.^32^

While the overall function of interoception is to process internal parameters, interoception is not an isolated domain; interoception interacts with exteroception, cognition, and action to ensure the integrity of the organism is maintained.^18^ For example, the interoceptive sensation of hunger drives food-seeking behaviours (eg, foraging). Hunger states can also bias towards the perceptual processing of visual food cues^56^ and enhance the pleasantness ratings of food images.^48^ Thus, internal bodily signals shape cognition and stimulus processing, influence action, and “colour” affective state. The dynamics between interoceptive signalling and the influence on cognition, affect, and action are observed through research showing how visceral oscillations in brain can alter stimulus processing. For example, when stimuli are processed in time with cardiac systole (during the cardiac ejection period, when baroreceptors are activated, and the brain processes how fast and hard the heart is beating), relative to cardiac diastole (when baroreceptors are quiescent, and this heart–brain channel is quiet) stimulus processing is altered; fear intensity ratings are augmented,^24,25^ memory encoding is impaired,^23^ and the perception of time contracts.^5^ These moment-to-moment processing changes coupled to the cardiac cycle (which could be conceptualised as an “artefact” of the system) mirror changes that can occur in more prolonged states of autonomic change, which may serve an adaptive function. For example, stress can increase fear reactivity,^43^ impair memory encoding to optimise other types of processing,^45^ and change time perception.^53^ However, unlike stress that changes multiple bodily systems and cognitive processes, determining the moment-to-moment changes in cognitive and affective processing tied to visceral oscillations in brain can provide more specific mechanistic insights into how interoceptive signalling directly alters the way we process, perceive, and act.

Physiological drive is fundamental to survival, and thus interoceptive signalling is closely attuned with goal-directed actions. Vagal afferent stimulation can evoke changes in brain function, leading to changes in goal-directed behaviour such as reward seeking.^51^ Visceral oscillations, such as the cardiac cycle, have been shown to impact action tendencies; active sampling in visual search is coupled to the cardiac cycle,^22^ and spontaneous movements are also more likely to occur at specific points in the cardiac cycle.^39^ These findings are in line with research detailing that systolic cardiac signals have a facilitatory effect on motor excitability.^2^ The body can also change visceral oscillations (ie, the afferent signals themselves) to prioritise rapid action.^3^ Motivated action is intrinsically coupled to bodily state, and interoceptive pathways can help elucidate the mechanisms through which internal signals may drive or inhibit certain action tendencies. Together interoceptive research details how urge to act and action timing can be influenced by interoceptive signals.

The study of interoception also reveals individual differences in body-to-brain processing. Individuals differ extensively in their accuracy (or “precision”) to detect internal bodily signals. The insula is a key interoceptive “hub” in the brain, and these structural and functional individual differences are important for understanding the mechanisms underlying differences in interoceptive profiles.^12^ Reduced amplitude of interoceptive signals in brain, such as the heartbeat-evoked potential, is observed in individuals with chronic emotional instability, such as borderline personality disorder.^37^ Thus, markers of body-brain integration, such as the heartbeat-evoked potential, can offer dispositional insights into the nature (and efficiency) of how the brain processes interoceptive signals, with implications for the regulation of internal states.

Attention to internal signals, such as heartbeats, can also amplify their neural representation, as observed via the augmentation of the heartbeat-evoked potential in brain when attention is directed to the heart.^41^ This principle has implications for symptom perception, where attention to internal signals may increase their neural dominance with implications for the strength with which they are processed. Attention oscillates between internal and external processing; when the brain is occupied by the processing of internal bodily signals, exteroceptive processing is disrupted. Cardiac signals heighten somatosensory thresholds^1^ and dampen awareness of exteroceptive visual cues within the environment.^44^ Flotation tanks that induce exteroceptive sensory deprivation enhance interoceptive awareness.^19^ Conversely, when attention is dominated by external cues, interoceptive processing is attenuated. Understanding the interoceptive dynamics of switching between physical and environmental cues, or between cognition and perception, can provide mechanistic insights into the engagement and disengagement of attention from salient information.^8,52^

Strong individual differences in interoceptive accuracy exist, supported by corresponding structural and functional individual differences in the brain. Individual differences in the nature of body–brain integration map onto differences in affective style, such as a tendency for chronic emotional dysregulation. Attention oscillates between internal and external processing; when cues in the environment dominate attention, interoceptive signals are less likely to break into conscious awareness.

**Figure 1.**
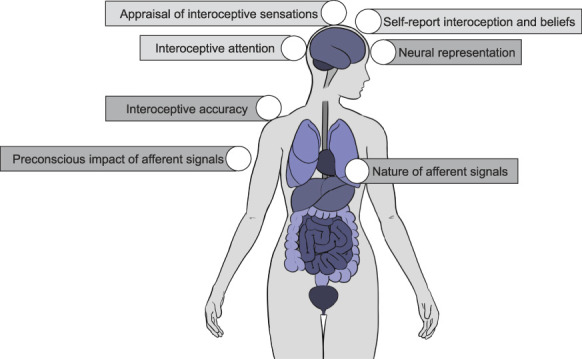
Interoception can be delineated across different hierarchical levels including the nature of afferent signals, how afferent signals can alter different type of processing (“preconscious impact of afferent signals”), the accuracy with which people can detect interoceptive sensations (“interoceptive accuracy”), the neural processing of interoception and body-brain integration (“neural representation”) and higher order measures pertaining to interoceptive beliefs, interoceptive attention and the appraisal of interoceptive sensations.

Together, these findings and observations highlight that interoception is not a unitary process but has different hierarchal levels each with meaningful and discernible effects on cognition, affect, and behaviour: (1) the nature of afferent signals (eg, shown by changes in heart rate [HR] and heart rate variability [HRV], parasympathetic tone), (2) the preconscious impact of afferent signals (eg, shown by cardiac cycle effects, which can influence signal processing including fear), (3) neural representation of interoceptive signals (eg, the heartbeat-evoked potential), (4) the accuracy or “precision” with which individuals can detect internal bodily sensations, (5) self-reported measures of interoception and interoceptive beliefs (which can differ from behavioural indices of interoceptive accuracy^26^), (6) attention to interoceptive signals, and (7) higher-order appraisal, attribution, and interpretation of these signals (Fig. 1). These levels are discussed in relation to pain below and detailed in Table 1.

**Table 1:** Dimensions of interoception and their implications for individual differences in pain and pain behaviour.

Nature of afferent signals	Individuals differ in the nature of their afferent signals such as HR and HRV.^11^ As these signals have implications for how pain is processed, this individual variation may be informative for understanding pain profiles, decision making, and affective responses to pain. A research programme into the nature of afferent signals and pain could assess multiple levels of autonomic change across different bodily axes to see whether they map onto individual pain profiles and intensity of pain perception. Longitudinal studies would help differentiate between cause and effect for afferent signals and pain perception.
Preconscious impact of afferent signals	Afferent signals can attenuate pain processing.^24,29^ Individual differences in the efficacy of this may have implications for individual differences underlying pain and pain behaviour. Future studies can ascertain using how afferent signals may shape the nature of pain processing in different individuals with chronic pain and heightened pain sensitivity, using causal techniques such as cardiac-cycle effects on pain processing^29^ (ie, experimental pain) and manipulations to induce autonomic change to observe shifts in pain perception (ie, related to chronic pain). Preconscious preparation for flight or avoidance of the threat of pain can be observed in the afferent signal, which may also have implications for the processing of pain.
Neural representation of interoceptive signals	Neural indices of body–brain integration (such as the heartbeat-evoked potential) map onto individual differences in pain profiles. Individual differences in the functional and structural architecture in brain underlying interoception may also have implications for pain processing. Future studies should be designed to determine causality of this body–brain signal.
Interoceptive accuracy	Individuals differ in the accuracy with which they can detect internal bodily sensations. This interoceptive precision maps onto pain thresholds, may differ in individuals with chronic pain conditions, and may protect against high impact chronic pain. Longitudinal studies to determine whether interoceptive accuracy profiles predict the likelihood of chronic pain, in addition to experimental treatment approaches that train interoceptive accuracy to determine reductions in the experience of pain, can help ascertain the nature of causality between interoceptive precision and pain.
Self-report measures of interoception and interoceptive beliefs	Self-reports about interoceptive accuracy do not always correspond with laboratory measures of interoceptive accuracy^26^ with implications for the report of bodily events as symptoms.^26^ This lack of concordance can also predict anxiety and fear of bodily events.^27^ Given the extent of experiential avoidance and fear of pain in chronic pain populations, exploring beliefs about bodily signals and their effect on interoception and subsequent behaviour could be informative.^49,54^
Interoceptive attention	Attending is a function of the demands of motivated action and the demands of central bodily signals. The dynamic between interoceptive and exteroceptive processing, including underlying neural “capture” by interoceptive signals, may provide insights into why body-based processing and pain may dominate attention in some individuals. For example, future research can be done that further explores how the dominance of interoceptive signals represented in brain may causally capture attention towards the body. Similarly, a focus on behavioural coherence and how incoherence or rupture in behaviour by dominant interoception is managed will be instructive.^14^
Appraisal of interoceptive sensations	Once selected, appraisal of the threat value of pain becomes critical, as does the wider concordance with a common-sense model of health and illness that dominates, eg, in cancer pain.^30^ The extent to which the appraisal process of identification, labelling, causally attributing, seeking further evidence, and then planning for action is captured in chronic pain as worry.^4,17^ Instructive would be a closer examination of the effect of worry (and other appraisal styles) on interoceptive accuracy, neural representation, and afferent drive.

HR, heart rate; HRV, heart rate variability.

## 3. Interoception and pain

Pain functions to interrupt current attention, interfere with current goals, and over time alters identity.^14,16,50^ Pain can usefully be considered a rupture, a breach in the harmonious coupling of body and brain, evidenced in disrupted behaviour. Pain functions to impose novel priorities of escape, avoidance, and alarm and activates defence systems more broadly.^6,46^ As interoception is the primary mechanism of homeostasis and behavioural coherence in the presence of the threat of harm, it is, therefore, implicated in any study of the protective function of pain.

There is a rich literature on the consequences of pain once it reaches attention and its cognitive fate,^7–9,35^ but less on how it achieves that dominance, capturing and fixing attention. There is, however, only a small track of research on interoception and pain.

### 3.1. Nature of afferent signals

Interoceptive signals provide an “internal context” that shape how stimuli are processed. These experiments have been done extensively outside the field of pain, as detailed above, and empirical work has also demonstrated their relevance to pain. Increased parasympathetic tone is associated with higher pain inhibition capacity, an effect, along with baroreflex sensitivity, that predicts postoperative pain.^38^ In a meta-analysis of experimental pain studies in nonclinical individuals, an association between HRV (HRV; an index of cardiac parasympathetic tone) and pain was confirmed, yet “future studies are also needed in clinical samples.”^21^ Heart rate variability indexes the dynamic regulation of cardiovascular arousal in response to phasic feedback from arterial baroreceptors. Baroreceptors are activated phasically with each heartbeat at cardiac systole; this interoceptive signal can inhibit acute pain reflexes, neural responses, and subsequent pain experience, yet amplify the processing of threatened pain^28^ (ie, providing evidence to support preconscious impact of afferent signals on the perception of pain). Individual differences in autonomic profile (eg, HRV, extent of parasympathetic tone at rest) may help understand similar variability in pain behaviour, including environmental responsivity such as to acute stress.^31^ Further understanding the nature of these lower-level interoceptive signals, and how they interact with other dimensions of interoception, such as body–brain integration, could yield new insights.

### 3.2. Neural representation of interoceptive signals

Typically, in body and brain, signals in relation to pain are investigated separately; however, it is possible to integrate them. For example, heartbeat-evoked potentials (HEPs), an electrophysiology brain signal reflecting cortical processing of heartbeats, are relevant to pain.^40^ Preliminary research using evoked pain in healthy individuals suggests that increased levels of body–brain integration are associated with *a higher* threshold for pain, and that HEP amplitude is inversely related to pain ratings.^47^ This initial evidence links pain states to a *rupture of body–brain integration*. However, in chronic pain patients, measures of body–brain integration and their implications remain poorly understood. While strong individual differences in visceral-brain integration are observed across the population, it is unknown how this relates to chronic pain conditions.

### 3.3. Interoceptive accuracy

Heightened interoceptive accuracy within the cardiac domain is associated with greater experimental pain sensitivity; specifically, pain thresholds are reduced in individuals who can detect their own heartbeats with increased precision at rest.^42^ For individuals without chronic pain, sensitivity to internal signals may augment pain sensations. In contrast, interoceptive precision within the cardiac domain is *reduced* in some patients with chronic pain,^13^ suggesting a dampening of internal signals because of persistent pain. It is not clear how far a lack of interoceptive precision or tendency to attenuate pain is phenotypical in the onset or maintenance of high impact chronic pain. Conversely, the integrity of body–brain mapping, marked by an increased precision to internal sensations, may be a protective factor against high impact chronic pain development. Lower interoceptive accuracy in fibromyalgia is associated with greater pain-related affect and reactions, detailing how interoceptive profiles may also have implications for the emotional processing of pain.^10^ Given that interoceptive accuracy is malleable, it provides a promising treatment target. Indeed, training somatosensory precision (eg, tactile acuity on skin) has been shown to significantly reduce pain experience in a number of complex chronic pain conditions, including phantom limb pain,^20^ nonspecific low back pain,^55^ and complex regional pain syndrome.^36^ There may be promise in determining the potential causal role of interoceptive accuracy in pain states and transitions, and by extension whether interoceptive training could be a useful mechanistic treatment target, analogous to precision training in the somatosensory modality.^15,16^

## 4. Embracing interoception in pain mechanism research

Arguably interoception is a “hidden” level of mechanism that has, to date, largely been neglected from pain research. In the decade of the brain, our focus shifted from the psychological study of behaviour to the biological study of brain, bypassing the body.^34^ Interoception positions “the internal body” and body–brain integration as central to coherent action. This focus can offer a bridge between different languages, methods, and approaches in pain science and could provide novel insights and empirical direction. Building on the foundation of earlier studies,^11,25^ we propose a comprehensive direction for a programme of study, with multiple mechanisms of mind–body integration, disintegration, and reintegration at its centre.^32,49^

A hierarchical model of interoception could reveal novel pain mechanistic insights. Contemporary models of interoception incorporate these different hierarchical levels of processing, including the nature of afferent signals, their neural processing, the accuracy with which they can be sensed, and higher-order levels pertaining to attention to internal bodily signals and their appraisal.^49^ These different interoceptive levels have selective and interacting implications for the preconscious processing of nociceptive signals and the perception of pain, as outlined in Table 1. An interoceptive investigation of body–mind rupture in pain requires analysis at all 7 levels to build what we call a pain interoceptive profile.

Some aspects of this profile will be more important to different people and at different times. For example, when acute pain is planned and controlled—as in procedural or postoperative settings—one can also strongly influence beliefs, attention, and interpretation. However, when pain is characterised by randomness, uncertainty, and uncontrollable impact, it will be harder to use higher order strategies and altering early interoceptive processes may be more possible. Different interoceptive mechanisms may also be more relevant to certain chronic pain conditions. Carefully planned experiments, such as longitudinal studies, are required to determine causality; changes in cardiac perceptual precision may be associated with pain behavioural differences, but is that a direct effect by common processes of interoception, or are both independently dependent on differences in efferent autonomic activity/tone? Finally, the autonomic system is of course an integrated system that bi-directionally interacts with pain; autonomic signals can both change in response to acute pain^33^ as well as shape the perception of pain (as detailed by causal and controlled cardiac cycle experiments).

In conclusion, we argue for a timely adoption of a thoroughgoing investigation of interoceptive mechanisms in pain, in particular in the onset, maintenance of, or recovery from high impact chronic pain. There is a foundation to build from: we have a fast-growing field in cognate areas such primary mental health disorders; we have many studies of somatic attending, interpretation, and higher order beliefs; and we have a small number of pain investigations of specific interoceptive measures such as cardiovascular variability. We can build on this foundation by considering pain at multiple levels of interoceptive influence, with a view to identifying novel targets for therapeutic intervention.

## Conflict of interest statement

The authors have no conflicts of interest to declare.
